# Gold Nanoparticles Encapsulated Resveratrol as an Anti-Aging Agent to Delay Cataract Development

**DOI:** 10.3390/ph16010026

**Published:** 2022-12-25

**Authors:** Qifang Chen, Peilin Gu, Xuemei Liu, Shaohua Hu, Hong Zheng, Ting Liu, Chongyi Li

**Affiliations:** 1 Department of Ophthalmology, Daping Hospital, Army Medical University, Chongqing 400042, China; 2 Department of Thoracic Surgery, Xinqiao Hospital, Army Medical University, Chongqing 400037, China

**Keywords:** gold nanoparticles, resveratrol, reactive oxygen species, cellular senescence, cataract

## Abstract

Nanoparticle-based drug delivery systems, which can overcome the challenges associated with poor aqueous solubility and other harmful side effects of drugs, display potent applications in cataract treatment. Herein, we designed a nanosystem of gold nanoparticles containing resveratrol (RGNPs) as an anti-aging agent to delay cataracts. The spherical RGNPs had a superior ability to inhibit hydrogen peroxide-mediated oxidative stress damage, including reactive oxygen species (ROS) production, malondialdehyde (MDA) generation, and glutathione (GSH) consumption in the lens epithelial cells. Additionally, the present data showed that RGNPs could delay cellular senescence induced by oxidative stress by decreasing the protein levels of p16 and p21, reducing the ratio of BAX/BCL-2 and the senescence-associated secretory phenotype (SASP) in vitro. Moreover, the RGNPs could also clearly relieve sodium selenite-induced lens opacity in a rat cataract model. Our data indicated that cell senescence was reduced and cataracts were delayed upon treatment with RGNPs through activating the Sirt1/Nrf2 signaling pathway. Our findings suggested that RGNPs could serve as an anti-aging ingredient, highlighting their potential to delay cataract development.

## 1. Introduction

Cataracts, a primary cause of blindness worldwide, seriously influence the quality of human life [1,2]. According to the location of the opacification of the eye lens, they are classified into cortical, nuclear, and posterior subcapsular [3]. Currently, the only available treatment for cataract patients is removing the opacified lens, replacing it with an artificial one, and wearing an appropriate corneal contact lens after an operation [4,5]. However, there are drawbacks caused by the technique, including postsurgical inflammation and implant disintegration. Thus, less invasive techniques and better drug delivery approaches are needed.

The advancements in nanotechnology can provide an opportunity for the non-invasive treatment of cataracts. Nanomachines can aid surgical techniques and the implementation of new medical therapies [6]. Nanostructures improve biocompatibility because of their small size and tunability to selectively deliver drugs to the specified location [7]. As is known, the classical drugs for cataract treatment are either systemic or topical because of the limitations in the drug’s delivery and bioavailability, mainly due to numerous barriers that isolate and protect the eye from the external environment. Nanotechnology has already played a vital role in developing microneedles, biosensors, and diagnostic labeling strategies in ophthalmology [8]. Therefore, the combination of drugs and nanostructures may improve the overall therapeutic effect of cataracts.

Resveratrol (3,5,4-trihydroxy-trans-stilbene, Res) is a natural polyphenol found in plants such as peanuts, grapes, and strawberries [9,10]. Recently, many studies have reported that Res possesses anti-inflammatory, anti-cancer, and anti-bacterial properties [11,12,13]. Intriguingly, the ability of Res to induce antioxidant formation in many cell types has validated its anti-aging potential, which has, in turn, attracted extensive attention in the research community. Oxidative stress leads to the extreme rise of reactive oxygen species (ROS), which can accelerate cell aging and damage DNA, RNA, and proteins [14]. Cellular senescence, a cell fate that entails essentially irreversible replicative arrest, is a leading cause of cataracts [15]. Senescence induced by UV radiation, oxidation, and truncation cause the most common modifications in lens proteins, affecting cataract formation. Additionally, studies have demonstrated that Res could prevent oxidative stress and cellular senescence by activating the silent information regulator 1 (Sirt1)/nuclear factor erythroid 2-related factor 2 (Nrf2) pathways [16]. Gold nanoparticles (GNPs) are excellent biological carriers because of their good biocompatibility. Moreover, gold is also reported to be an anti-aging metal [17]. 

**Scheme 1:**
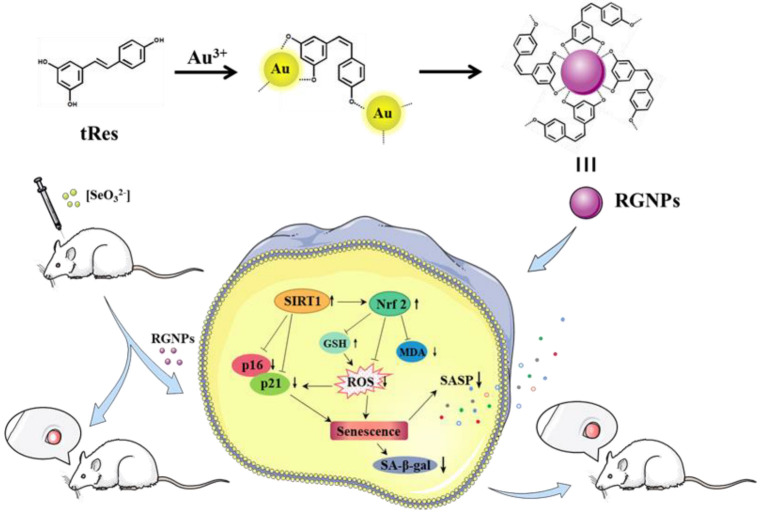
Illustration of the anti–aging potential of RGNPs to delay cataract development in vitro and in vivo.

Herein, we hypothesized that the gold nanoparticles encapsulated with resveratrol (RGNPs) would generate a better anti-aging effect than treatment with Res alone for slowing the progression of cataracts. To evaluate the anti-aging activity of RGNPs in vitro and in vivo, we comparatively investigated the effects of Res and RGNPs in lens epithelial cells and a rat cataract model. Our results highlight the inhibition of oxidative stress-mediated ROS production and the activation of the Sirt1/Nrf2 signaling pathway, which could further delay the cataract development (Scheme 1).

## 2. Results

### 2.1. Preparation and Characterization of RGNPs

**Figure 1:**
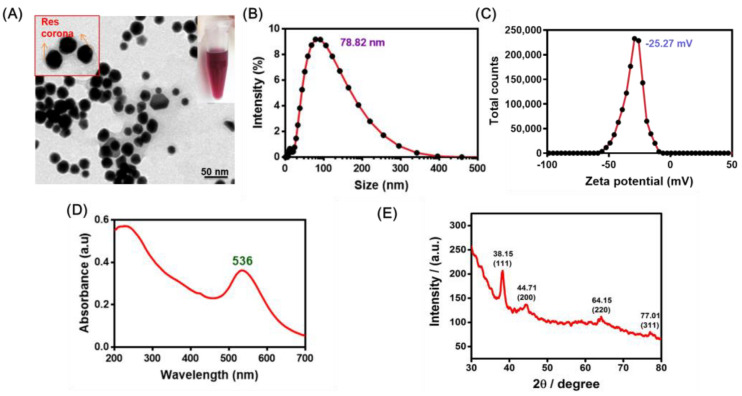
The characterization of gold nanoparticles containing resveratrol (RGNPs). (**A**) Transmission Electron Microscope (TEM) image of RGNPs. (**B**) The hydrodynamic size distribution of RGNPs. (**C**) The zeta potential of RGNPs. (**D**) UV–vis spectrum of RGNPs. (**E**) X-ray diffraction (XRD) pattern determining the crystalline nature of RGNPs.

RGNPs were obtained with a molar ratio of 1:2 (Au/tRes) according to the previous study, wherein tRes was used as a potential reducing and stabilizing agent for GNP synthesis [18]. The red solution was observed intuitively to successfully prepare RGNPs. The Transmission Electron Microscope (TEM) images confirmed that the RGNPs were sufficiently mono-dispersed and spherical with a mean diameter of about 35 nm, and the corona around them elucidated the successful functionalization of GNPs through Res, as shown in Figure 1A. Further characterization of RGNPs by dynamic light scattering (DLS) provided the hydrodynamic size of 78.82 nm and the surface charge of −25.27 mV in Figure 1B,C, which indicated that RGNPs have good stability in water. In addition, the absorption maxima (λmax) measured by UV-visible absorption spectra due to Surface Plasmon Resonance (SPR) was observed at approximately 536 nm (Figure 1D), which further confirmed the formation of RGNPs. Furthermore, the X-ray diffraction (XRD) pattern presented four distinct characteristic peaks of crystalline RGNPs at 38.15º (111), 44.71º (200), 64.15º (220), and 77.01º (311), which indicated the prepared RGNPs were crystalline in nature (Figure 1E). All these data indicated that RGNPs were successfully synthesized.

### 2.2. Cellular Uptake of RGNPs

**Figure 2:**
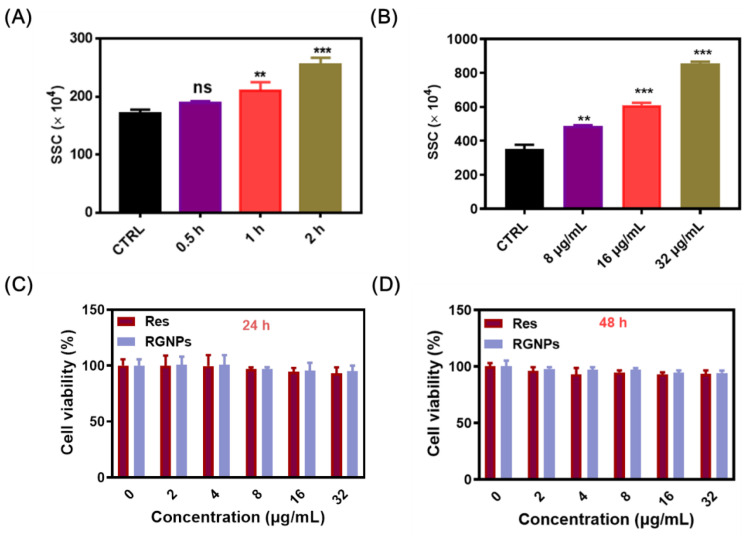
Cellular uptake in vitro. (**A**) Side scatter (SSC) values of HLECB3 cells treated with RGNPs with different incubation times via flow cytometry (FCM) analysis. (**B**) SSC values of HLECB3 cells treated with different concentrations of RGNPs via FCM analysis. (**C**) Cell viability of HLECB3 cells treated with different concentrations of RGNPs for 24 h. (**D**) Cell viability of HLECB3 cells treated with different concentrations of RGNPs for 48 h. ** and *** indicate *p* < 0.01 and *p* < 0.001; ns indicated statistically insignificant.

The RGNPs should be efficiently internalized to exert the antioxidant effects by lens epithelial cells. In the uptake experiment, the side scatter (SSC) of HLECB3 cells in the flow cytometer detection chart showed a concentration and time-dependent increase, indicating that RGNPs could be successfully internalized into HLECB3 cells, as shown in Figure 2A,B. To determine the cytotoxicity after internalization, we found that there was almost no damage to cells with the increase in the concentration of RGNPs (2, 4, 8, 16, 32 μg/mL) and incubation time (24 and 48 h) through a CCK8 assay, which indicated RGNPs could be available for subsequent study because of the biocompatibility of RGNPs.

### 2.3. Antioxidant Effect of RGNPs in HLECB3 Cells

**Figure 3:**
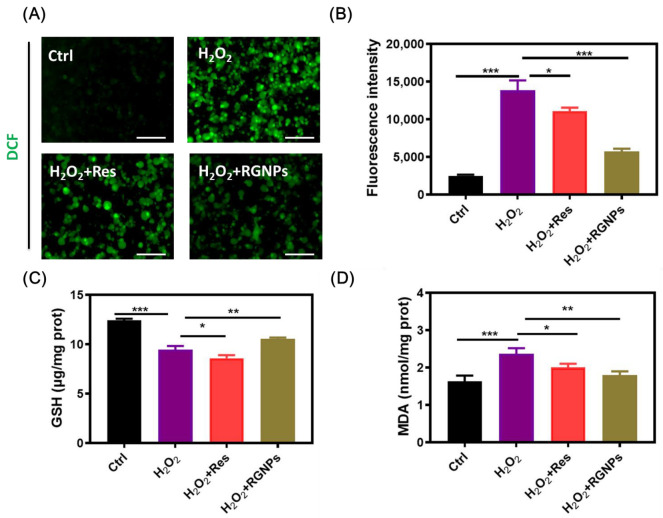
Anti–oxidant effect of RGNPs. (**A**) Confocal laser scanning microscope (CLSM) images of HLECB3 cells stained by 2,7-Dichloro-fluorescein diacetate (DCFH–DA, ROS probe) after treatment with different formulations (Ctrl, H_2_O_2_, H_2_O_2_ + Res, H_2_O_2_ + RGNPs) for 12 h; the bar was 50 μm. (**B**) Fluorescence intensity of HLECB3 cells stained by a DCFH–DA probe after treatment with different formulations for 12 h. (**C**) Intracellular glutathione (GSH) level of HLECB3 cells after treatment with different formulations for 24 h. (**D**) Intracellular malondialdehyde (MDA) level of HLECB3 cells after treatment with different formulations for 24 h. *, ** and *** indicate *p* < 0.05, *p* < 0.01 and *p* < 0.001.

Res possess anti-carcinogenic, anti-inflammatory, anti-bacterial, and antioxidant properties [19]. Thus, we examined the intracellular generation of ROS from RGNPs using 2,7-Dichloro-fluorescein diacetate (DCFH-DA) as a fluorescent probe to evaluate whether RGNPs have better antioxidant activity than Res. As shown in Figure 3A,B, strong green fluorescence was observed in H_2_O_2_-incubated HLECB3 cells, which implied that the H_2_O_2_ induction group contained abundant intracellular ROS concentration because of oxidative stress injury. RNGPs treatment significantly decreased the green fluorescence intensity of HLECB3 cells compared to treatment with the Res group, suggesting a superior antioxidant capacity of RGNPs to inhibit ROS generation. In addition, the effect of Res and RGNPs on intracellular glutathione (GSH) and malondialdehyde (MDA) levels revealed a decrease and an increase, respectively, in the H_2_O_2_ group compared to the control group. Treatment with RGNPs significantly increased the GSH levels and reduced the MDA levels compared to treatment with Res in Figure 3C,D. These results demonstrated that RGNPs had superior antioxidant effects to prevent oxidative stress damage than single Res treatment.

### 2.4. Anti-Aging Properties of RGNPs in HLECB3 Cells

**Figure 4:**
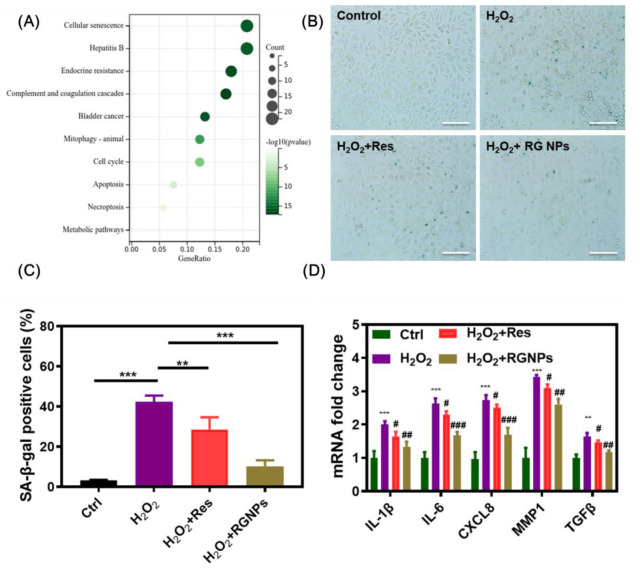
Anti–aging effect of RGNPs. (**A**) Gene Expression Omnibus (GEO) analysis of Res involved function enrichment (GSE184636). (**B**) Representative images of senescence-associated-β-galactosidase (SA–β–gal) staining in HLECB3 cells after treatment with Ctrl, H_2_O_2_, H_2_O_2_ + Res, H_2_O_2_ + RGNPs; the bar was 50 μm. (**C**) Quantitation of the SA–β–gal–positive cells. (**D**) mRNA fold change of senescence-associated secretory phenotype (SASP) factors (IL–1β, IL–6, CXCL8, MMP1 and TGFβ) in HLECB3 cells treated with Ctrl, H_2_O_2_, H_2_O_2_ + Res, H_2_O_2_ + RGNPs. ** and *** indicate H_2_O_2_ vs. Ctrl; #, ## and ### indicate H_2_O_2_ + Res or H_2_O_2_ + RGNPs vs. H_2_O_2_.

Res, an antioxidant polyphenol, has been the subject of intense interest recently because the ability of antioxidants in many cell types has confirmed the anti-aging properties of Res [20]. Besides, we found that cellular senescence could be enriched after treatment with Res from the Gene Expression Omnibus (GEO) dataset (GSE184636) in Figure 4A. To confirm the senescence, we measured senescence-associated-β-galactosidase (SA-β-gal) activity, a senescence biomarker, in HLECB3 cells. After adding H_2_O_2_, 42.4% of treated cells were SA-β-gal-positive, while 3.2% of control cells were SA-β-gal-positive; 28.6% and 10.2% SA-β-gal-positive cells were observed in Res and RGNPs, respectively, indicating the excellent anti-aging effect of RGNPs compared to Res (Figure 4B,C). Senescence-associated secretory phenotype (SASP), a series of inflammatory cytokines, chemokines, growth factors, and proteases secreted by senescence cells, is a feature of senescence [21]. To evaluate SASP, expressions of (IL-1β, IL-6, CXCL8, MMP1 and TGFβ) were analyzed in HLECB3 cells. As shown in Figure 4D, expression levels of SASP were significantly higher in the H_2_O_2_ addition group while lower in RGNPs than in Res treatment, confirming that RGNPs could inhibit cellular senescence induced by H_2_O_2_. 

### 2.5. RGNPs Inhibited Cellular Senescence via Activation of the Sirt1/Nrf2 Pathway In Vitro

**Figure 5:**
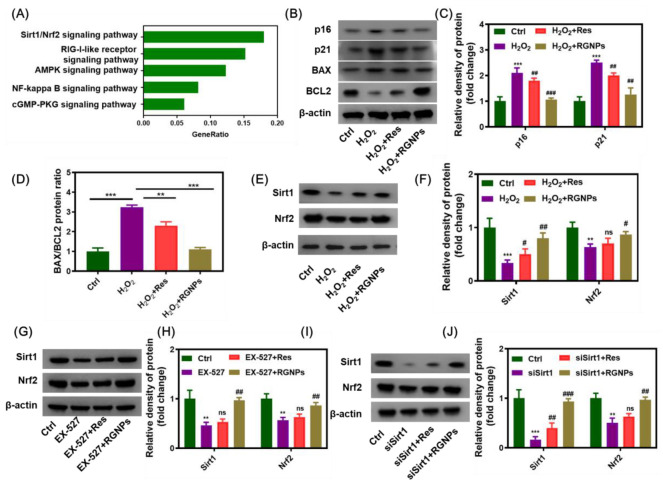
RGNPs inhibited cellular senescence via activation of Sirt1/Nrf2 pathway in vitro. (**A**) GEO analysis of Res involved signaling pathway (GSE184636). (**B**) Senescence-related proteins by H_2_O_2_, H_2_O_2_ + Res and H_2_O_2_ + RGNPs treatment in HLECB3 cells, such as p16, p21, BAX and BCL2 were determined by western blotting. (**C**) Relative density of proteins (p16 and p21) by H_2_O_2_, H_2_O_2_ + Res and H_2_O_2_ + RGNPs treatment in HLECB3 cells. (**D**) BAX/BCL2 protein ratio by H_2_O_2_, H_2_O_2_ + Res and H_2_O_2_ + RGNPs treatment in HLECB3 cells. (**E**) Sirt1 and Nrf2 proteins by H_2_O_2_, H_2_O_2_ + Res and H_2_O_2_ + RGNPs treatment in HLECB3 cells were determined by western blotting. (**F**) Relative density of proteins (Sirt1 and Nrf2) by H_2_O_2_, H_2_O_2_ + Res and H_2_O_2_ + RGNPs treatment in HLECB3 cells. (**G**) Sirt1 and Nrf2 proteins by EX-527 (Sirt1 inhibitor), EX-527 + Res and EX-527 + RGNPs treatment in HLECB3 cells were determined by western blotting. (**H**) Relative density of proteins (Sirt1 and Nrf2) by EX-527, EX-527 + Res and EX-527 + RGNPs treatment in HLECB3 cells. (**I**) Sirt1 and Nrf2 proteins by siSirt1, siSirt1 + Res and siSirt1 + RGNPs treatment in HLECB3 cells were determined by western blotting. (**J**) Relative density of proteins (Sirt1 and Nrf2) by siSirt1, siSirt1 + Res and siSirt1 + RGNPs treatment in HLECB3 cells. ** and *** indicate H_2_O_2_ or EX-527 or siSirt1 vs. Ctrl; #, ## and ### indicate H_2_O_2_ + Res, EX-527 + Res, siSirt1 + Res or H_2_O_2_ + RGNPs, EX-527 + RGNPs, siSirt1 + RGNPs vs. H_2_O_2_, EX-527, siSirt1; ns indicated statistically insignificant.

Western blotting was performed to understand how RGNPs modulate H_2_O_2_-induced ROS production and cellular senescence. The effects of Res were mediated via the activation of the Sirt1 signaling pathway, which has been proven to prevent cell aging. And Res, an Nrf2 activator, could also prevent aging and oxidative stress [22]. Additionally, we investigated whether the Sirt1/Nrf2 signaling pathway could be enriched after treatment with Res from the GEO dataset (GSE184636) in Figure 5A. Therefore, we hypothesized that RGNPs also inhibited cellular senescence by activating this pathway. As shown in Figure 5B–D, expression levels of senescence markers [p16INK4A, p21, BAX/BCL2 ratio] were significantly lower in RGNPs than in Res. In addition, the expression of Sirt1 and Nrf2 was reduced after the addition of H_2_O_2_ compared to the control group. Treatment with RGNPs significantly increased the expression of Sirt1 and Nrf2 more than Res treatment, which indicated RGNPs inhibited cellular senescence induced by H_2_O_2_ via activation of the Sirt1/Nrf2 pathway (Figure 5E,F). Moreover, this result was further confirmed by adding a Sirt1 inhibitor (EX-527) and transfection of Sirt1 siRNA (siSirt1), respectively. As shown in Figure 5G–J, the expression of Sirt1 and Nrf2 was decreased by EX-527 and siSirt1 treatment, while RGNPs could increase the expression of Sirt1 and Nrf2 more than the Res group. 

### 2.6. RGNPs Delayed Cataract Development by Inhibiting Lens Senescence In Vivo

**Figure 6:**
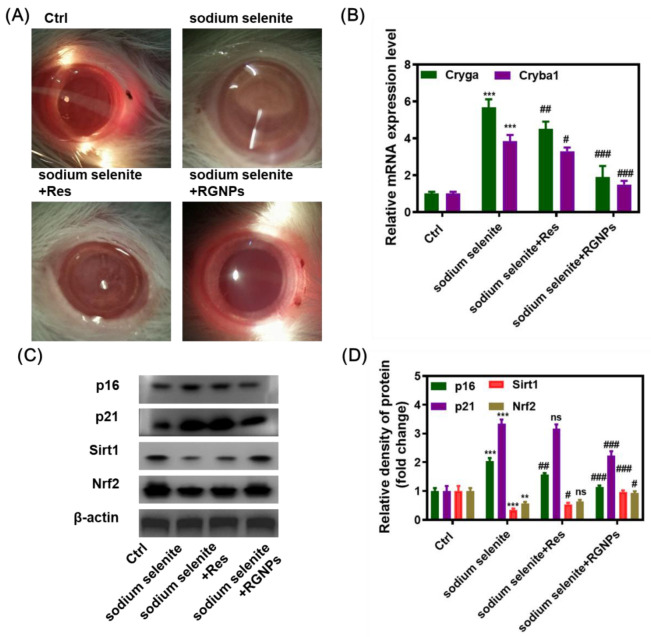
RGNPs delayed cataract development by inhibiting lens senescence in vivo. (**A**) Photographs of lens opacity in rat eyeball after treatment with sodium selenite, sodium selenite +Res and sodium selenite +RGNPs. (**B**) Relative mRNA expression levels of Cryga and Cryba1 in rat lens treated with sodium selenite, sodium selenite +Res and sodium selenite +RGNPs. (**C**) p16, p21, Sirt1 and Nrf2 proteins by sodium selenite, sodium selenite +Res and sodium selenite +RGNP treatment in rat lens were determined by western blotting. (**D**) Relative density of proteins (p16, p21, Sirt1 and Nrf2) by sodium selenite, sodium selenite +Res and sodium selenite +RGNPs treatment in rat lens. ** and *** indicate sodium selenite vs. Ctrl; #, ## and ### indicate sodium selenite +Res or sodium selenite +RGNPs vs. sodium selenite; ns indicated statistically insignificant.

Studies have reported that the development of senescence in lens epithelial cells is essential for cataracts. Hence, we investigated whether RGNPs can delay this development in rat cataract models. After treatment with sodium selenite and Res/RGNPs, the eyes of rats were dilated to observe the lens opacity (control, sodium selenite, Res, and RGNPs groups) with a slit lamp and anterior segment photography. As shown in Figure 6A, the lens in the control group was transparent, while it was turbid in the sodium selenite group, indicating that the cataract establishment was successful. After 14 days of Res and RGNPs treatment, the RGNPs treatment group reduced the lens opacity significantly more than the Res treatment group, which confirmed that RGNPs could delay cataract development by inhibiting the lens opacity in vivo.

Furthermore, we analyzed the mRNA expression levels of two kinds of lens proteins (Cryga and Cryba1) in rat lenses through a Real-time quantitative PCR (RT-qPCR) assay. As shown in Figure 6B, the expression levels of Cryga and Cryba1 in the cataract model group were up-regulated. At the same time, these were significantly down-regulated after RGNP treatment compared with the Res group. To examine whether RGNPs could delay the development of cataracts by inhibiting lens senescence, we removed the eyeball and extracted the proteins for Western blotting. As shown in Figure 6C,D, senescence markers (p16 and p21) were significantly up-regulated in the cataract model group, while they were reduced after treatment with RGNPs compared to Res treatment. Consistent with the in vitro experimental results, the protein expression levels of Sirt1 and Nrf2 were significantly decreased in the cataract group and significantly increased in the RGNP groups compared to the Res group. These results indicated that RGNPs delay cataract development by inhibiting lens senescence via the activation of the Sirt1/Nrf2 pathway in vivo. 

## 3. Discussion

In this study, we prepared an anti-aging drug carrier with higher bioavailability, RGNPs, to investigate the effect of delaying cataract in vitro and in vivo and further explore its mechanisms. Resveratrol is a biocompatible polyphenol molecule with significant pharmacological effects. However, its poor bioavailability and metabolic stability have limited its clinical application. Prior studies have looked at improving the bioavailability and safety of Res through Res-Au complexes, which explains the reason behind tRes in solution [23]. tRes dissociated into hydroxyl hydrogen protons and phenol hydroxyl oxygen negative ions, and then the negative charge caused racemization of tRes. When Au^3+^ was added, it was reduced to Au because of the ability to accept electrons of oxygen anion, and finally, tRes was oxidized to form isomer cRes [18,24]. Our experiments also proved that the prepared RGNPs were highly hydrophilic and could be well internalized by HLECB3 cells. These results showed that Res could effectively decrease the degree of cellular oxidative damage, restore the level of reducing substance GSH, and reduce the ratio of SA-β-gal-positive cells and the expression levels of SASP factors caused by H_2_O_2_ to inhibit cellular senescence.

In contrast, RGNPs showed more substantial antioxidant capacity and anti-aging effect than Res. We speculated that this might be attributed to two reasons: 1. Improvement of hydrophobicity and bioavailability of Res; 2. The beneficial effects of Au NPs, as they contain specific anti-inflammatory, anti-bacterial, and other pharmacological effects and are widely used in drug delivery and non-viral gene delivery [25,26]. These results suggested that RGNPs had excellent pharmacological effects and transformative potential. 

Cellular aging is a highly heterogeneous process, mainly induced by ROS-mediated oxidative stress [27,28,29]. With the increase in age, ROS gradually accumulates in cells, resulting in DNA damage which also blocks damaged nuclear and mitochondrial DNA repair mechanisms, thus leading to the instability of the cell genome, cell senescence, and age-related diseases [30]. In this study, we found that RGNPs could significantly reduce oxidative damage, including the production of ROS, the consumption of GSH, and the generation of MDA caused by H_2_O_2_ to inhibit cellular senescence such as the ratio of SA-β-gal-positive cells and the expression levels of senescence markers (p16, p21, BAX, BCL-2 and SASP) more than Res, which indicate that RGNPs can be used as an anti-aging agent.

During the pathogenesis of cataract development, lens epithelial cells are in a state of oxidative stress. Studies have found that Sirt1 could protect HLECB3 against oxidative stress by inhibiting p53-dependent apoptosis [31]. In addition, Sirt1 protein levels in various senescent cell models decreased gradually with the aging process, indicating that the loss of Sirt1 protein was specific to aging [29]. Resveratrol is currently recognized as a Sirt1 promoter and an activator of Nrf2 [22,24,32,33]. Similarly, analysis of GEO datasets (GSE184636) confirmed that the beneficial effects of Res depended on the activation of Sirt1/Nrf2. Compared with the Res treatment group, RGNP treatment could effectively increase the expression levels of Sirt1 and Nrf2 in the senescent cells, which confirmed that RGNPs inhibited cellular senescence through the Sirt1/Nrf2 pathway. 

ROS accumulation in the eyes leads to senescence, a crucial factor in age-related cataracts [34]. In vitro and in vivo experiments have shown that the development of cataracts correlates to reduced levels of glutathione, which could remove free radicals produced by the body and reduce the accumulation of oxidative stress substances in the body, at the center of the lens, and the oxidative modification of nuclear proteins [35]. Mice treated with RGNPs saw significantly improved lens opacity and showed relatively lower mRNA and protein levels of those proteins associated with lens (Cryga and Cryba1), aging (p16 and p21), and Sirt1 and Nrf2, compared with the cataract model group treated with sodium selenite. It is noteworthy that β-lens and γ-lens proteins, unlike α-lens proteins, have more sulfhydryl residues, which upon exposure produce more mixed disulfide and covalently cross-linked proteins, leading to lens opacity [36]. Hence, our findings indicated that the anti-aging effects regulated by Sirt1/Nrf2 for RGNPs were also effective in vivo. 

## 4. Materials and Methods

### 4.1. Materials

Resveratrol ≥99% and sodium tetrachloroaurate (III) dihydrate (NaAuCl4) ≥99% were purchased from Macklin Biochemical Co., Ltd. (Shanghai, China). GSH and MDA kits were purchased from Solarbio Bioscience & Technology Co., Ltd. (Beijing, China). Fetal bovine serum (FBS) and Dulbecco’s modified Eagle’s medium (DMEM) media were purchased from Thermofisher Scientific (Shanghai, China). Cell Counting Kit-8 (CCK-8) was from Bioground Bioscience & Technology Co., Ltd. (Chongqing, China). DCFH-DA was from Beyotime Biotechnology (Shanghai, China). EX-527 was obtained from Selleck (Shanghai, China). The primer for siSirt1 was designed by Sangon Biotech Co., Ltd. (Shanghai, China). Antibodies against p16, p21, β-actin, Sirt1, and Nrf2 were purchased from Abclonal Technology (Wuhan, China). BAX and BCL-2 were obtained from Abcam Plc (Shanghai, China). RT-qPCR amplification primers for GAPDH, IL-1β, IL-6, CXCL8, MMP1, TGFβ, Cryga, and Cryba1 were made by Sangon Biotech Co., Ltd. (Shanghai, China).

### 4.2. Synthesis of RGNPs

RGNPs was synthesized using the previous method with some modifications [18,37]. Briefly, 20 mL of 1 mM HAuCl_4_·3H_2_O was stirred at 25 °C for 5 min. To this, 2 mM of a tRes solution prepared in 2 mL of 0.02 M NaOH was introduced slowly under continuous stirring at 300 rpm for 3 h. Further, the RGNPs were collected by centrifuging at 14,500 rpm for 1 h followed by washing thrice to remove excess Res. 

### 4.3. Characterization of RGNPs

The morphology and size of RGNPs were determined through a TEM (FEI Technai 20U Twin). The mean hydrated particle size (nm) and zeta potential (mV) of RGNPs were determined by a DLS system (NanoZS; Malvern Instruments, Worcestershire, UK). The absorbance curve was determined by a UV-vis spectrometer (UV-1800, Shimadzu, Japan).

### 4.4. Cell Culture

The human lens epithelial cells (HLECB3) were obtained from Third Military Medical University (Chongqing, China). Cells were grown in DMEM supplemented with 10% FBS and in a humidified incubator supplemented with 5% CO_2_ at 37 °C. 

### 4.5. Establishment of Young Rat Cataract Model

Twenty eight-day-old rat pups were obtained from Daping Hospital (Chongqing, China) and randomly divided into control, cataract model, resveratrol, and RGNPs groups, with five rats in each group. The control group was injected with normal saline subcutaneously. The cataract model group was injected with 2 mM sodium selenite 20 μmol/kg subcutaneously at the neck and back from the 10th day of age, once every two days, three times consecutively. In addition to the injection of sodium selenite, resveratrol and RGNPs group were given 50 mg/kg by intraperitoneal injections once a day for 14 consecutive days from the 8th day of age (2 days ahead of schedule) [38]. When treatment culminated, the lens opacity of rats was observed by slit lamp photography. The eyeballs of the rats were then removed for subsequent experiments and analysis. 

### 4.6. Cellular Uptake In Vitro

HLECB3 cells were cultured in 6-well plates for 24 h, and RGNPs were added to the cell culture medium at different concentrations (8, 16, 32 μg/mL) and incubation times (0.5 h, 1 h, 2 h). After incubation, the cells were collected for SSC analysis of flow cytometry (FCM) (BD FACS Melody™).

### 4.7. Cell Viability Assay

Cell cytotoxicity was measured using a CCK8 kit. First, HLECB3 cells were seeded into 96-well plates for 24 h. Then, cells were co-incubated with different concentrations of Res and RGNPs for another 24 h and 48 h at 37 °C. After that, the culture medium was replaced by the 10% CCK-8 reagent and incubated at 37 °C for 1.5 h. Finally, the optical density was measured at 450 nm by a microplate reader (ELX-800).

### 4.8. Detection of the ROS

HLECB3 cells were pre-treated with Res (32 μg/mL) and RGNPs (32 μg/mL) for 6 h before exposure to 200 μM H_2_O_2_, followed by co-incubation for 24 h. Cells were co-incubated at 37 °C for another 30 min with a DCFH-DA probe after washing with 1XPBS. ROS levels in HLECB3 cells were observed using an inverted biological fluorescence microscope (Olympus, IX83). 

### 4.9. MDA Assay

HLECB3 cells were cultured in 100 mm Petri plates and then treated under different conditions (H_2_O_2_, Res, and RGNPs) for 24 h. According to the protein concentrations for each condition, the total MDA levels were measured and calculated by a commercial MDA kit. 

### 4.10. GSH Assay

Briefly, HLECB3 cells at a density of 1 × 10^6^ cells per well were seeded into 100 mm Petri plates. Cells were treated with H_2_O_2_, Res, and RGNPs for 24 h. As per the protein concentration of each formula, the total GSH levels were measured and calculated by a commercial GSH kit. 

### 4.11. SA-β-Gal Staining

The β-Galactosidase activity was evaluated by the Senescence Cells Histochemical Staining Kit (Sigma, St. Louis, MO, USA). Briefly, cells treated with H_2_O_2_, Res, and RGNPs were washed with phosphate buffer saline (PBS). 1 mL of fixation buffer was added and incubated with the cells for 8 min. Then cells were co-incubated with 1 mL of the staining mixture overnight at 37 °C (without CO_2_) to be stained blue. Blue-stained cells were then quantified relative to total cells, and percentage senescence was determined. 

### 4.12. Western Blotting Assay

HLECB3 cells were cultured in a 6-well plate for 24 h. Cells were treated with H_2_O_2_ (200 μM) or EX-527 (40 nM) or siSirt1 (50 nM), Res (32 μg/mL), and RGNPs (32 μg/mL) for 24 h. Cell lysates were collected and purified using centrifugation at 13,000 rpm at 4 °C for 15 min. Then, cell lysates were mixed with 5x loading buffer (20 μg/lane) and were resolved by electrophoresis on 4–20% prefabricated denaturing polyacrylamide gels (ACE Biotechnology, Xiangtan, China) and transferred to a nitrocellulose membrane. Subsequently, membranes were blocked with 5% fat-free milk and 0.1% Tween 20 in Tris-buffered saline for 1 h at 4 °C, and then incubated with a primary antibody (with 5% fat-free milk above). Further, the membranes were incubated with a horseradish peroxidase (HRP)-conjugated secondary antibody (1:4000) (Abclonal, Wuhan, China). Finally, the membranes were visualized using a ChemiDoc^TM^ Touch Imaging System (Bio-Rad Laboratories, Hercules, CA, USA).

### 4.13. RNA Extraction and RT-qPCR

Total RNA was extracted from HLECB3 cells and rat lens using Simply P Total RNA Extraction Kit (BioFlux, Beijing, China) and reverse-transcribed as cDNA with a PrimeScript RTPCR kit (Takara Biotechnology, Beijing, China). Then, RT-qPCR was performed for 40 cycles at 60 °C using a standard SYBR Green PCR kit (Roche, Shanghai, China). The 2^−ΔΔCt^ method was used to calculate the relative mRNA expression levels. 

### 4.14. Statistical Analysis

Statistical analysis was obtained by one-way analysis of variance (ANOVA). The graphs were prepared and analyzed through GraphPad Prism^®^ 7 software. The values of *p* < 0.05 (* or #), *p* < 0.01 (** or ##), and *p* < 0.001 (*** or ###) were regarded as significant. Unless otherwise stated, values are represented as mean ± SD.

## 5. Conclusions

The present data indicate that RGNPs could reduce the expression of senescence markers (p16, p21, BAX, BCL2, and SASP) and activate the Sirt1/Nrf2 signaling pathway in vitro and in vivo, so as to effectively inhibit the increase of ROS caused by oxidative stress and inhibit the senescence of lens epithelial cells. A rat cataract model further demonstrated the efficacy of RGNPs by inhibiting the lens opacity, indicating that gold nanoparticle-encapsulated resveratrol can significantly enhance the anti-aging effect of resveratrol in delaying the development of cataracts. Hence, RGNPs would be an excellent therapeutic ingredient for cataracts, providing a clinical theoretical basis for the treatment of cataract.

## Data Availability

Data is contained within the article.
